# LncRNA MIR4435-2HG triggers ovarian cancer progression by regulating miR-128-3p/CKD14 axis

**DOI:** 10.1186/s12935-020-01227-6

**Published:** 2020-05-01

**Authors:** Lijuan Zhu, Aihua Wang, Mei Gao, Xiaoyan Duan, Zehua Li

**Affiliations:** Department of Gynaecology and Obstetrics, The First People’s Hospital of Shangqiu, No. 292 Kaixuan South Road, Shangqiu, 476100 Henan China

**Keywords:** MIR4435-2HG, Ovarian cancer, miR-128-3p, CDK14

## Abstract

**Background:**

Accumulating studies showed that long noncoding RNAs (lncRNAs) played vital roles in cancer progression. LncRNA MIR4435-2HG was proved to act as an oncogene in various tumors. However, the underlying function of MIR4435-2HG in ovarian cancer (OC) remains unclear.

**Methods:**

The expression levels of MIR4435-2HG, miR-128-3p and cyclin-dependent kinase 14 (CDK14) were analyzed by quantitative real-time polymerase chain reaction (qRT-PCR). Cell proliferation and apoptosis in OC cells were detected by 3-(4, 5-dimethylthiazol-2-yl)-2, 5-diphenyltetrazolium bromide (MTT) assay and flow cytometric analysis, respectively. Transwell assay was applied to evaluate cell migration and invasion. Wound healing assay was performed to monitor the migration rate. Western blot assay was performed to detect the protein levels of Bcl-2, Cleaved PARP, E-cadherin, Vimentin and CDK14 in OC cells. The binding sites between miR-128-3p and MIR4435-2HG or CDK14 were predicted by online tool starBase and their relationship was confirmed by dual-luciferase reporter assay, RIP assay and pull-down experiment.

**Results:**

MIR4435-2HG and CDK14 were over-expressed in OC tissues and cells. Patients with high MIR4435-2HG expression had poorer overall survival (OS) than patients with low MIR4435-2HG expression. MIR4435-2HG knockdown inhibited proliferation, invasion and migration but induced apoptosis of OC cells via miR-128-3p/CDK14 axis. In conclusion, MIR4435-2HG knockdown suppressed the progression of OC cells through downregulating CDK14 expression by the promotion of miR-128-3p.

## Highlights

1. The target relationship between MIR4435-2HG and miR-128-3p is first disclosed.
2. The target relationship between miR-128-3p and CKD14 is first disclosed.
3. The effect of MIR4435-2HG/miR-128-3p/CKD14 axis on ovarian cancer is first presented.

## Background

Ovarian cancer (OC) is a universal tumor occurring in female that seriously threatens women’s physical and mental health. OC is the most deadly malignancy in gynecologic malignant tumors [1]. Most OC patients were diagnosed at advanced stages. With the progression of medical technology in recent years, some chemotherapeutic drugs were used to treat OC, but the prognosis of patients are still not satisfactory [2]. OC is the leading cause of cancer-associated mortality in women in worldwide and the five-year overall survival is less than 45% [3, 4]. Therefore, it is of important significance for clinical application to explore the mechanisms of OC progression.

Long non-coding RNAs (LncRNAs) are endogenous non-coding RNAs with the length of over 200 nucleotides, and ncRNA targeting protein-coding genes was reported to be an important regulatory molecule that can regulate the gene expression [5, 6]. A recent report suggested that lncRNA has a significant effect on the progression of numerous tumors and exerts its function as tumor suppressor or oncogene during tumorigenesis [7].

MIR4435-2HG is considered as a carcinogenic lncRNA in various cancers. For instance, up-regulated MIR4435-2HG interacted with poorer progression-free survival (PFS) and overall survival in colorectal cancer patients, and MIR4435-2HG was involved in the occurrence and evolution of colorectal cancer through regulating P38/MAPK and VEGF pathways [8]. MIR4435-2HG was overexpressed in hepatocellular carcinoma and promoted tumor cell proliferation by activating miR-487a [9]. A recent study presented that MIR4435-2HG was elevated in OC and the high expression of MIR4435-2HG triggered the migration and invasion of OC cells via activating TGF-β1 [10]. Moreover, miR-128-3p could participate in the progression of diver cancers through targeting different genes, and miR-128-3p regulated cell growth in breast cancer by targeting LIMK1 and overexpression of miR-128-3p in breast cancer patients presaged a good prognosis [11]. In glioma, the level of miR-128-3p expression was dramatically attenuated in glioma clinical tissues, and miR-128-3p regulated the progression of glioma via targeting NPTX1 and activating the IRS-1/PI3K/AKT signaling pathway [12]. Cyclin-dependent kinase 14 (CDK14) is a member of cyclin-dependent kinases, and a previous study demonstrated that CDK14 was augmented in the tissues and cells of OC [13].

In our study, we aimed to explore the role of MIR4435-2HG and its underlying mechanism in OC cells, and the results showed a novel mechanism of MIR4435-2HG/miR-128-3p/CKD14 axis and provided new potential therapeutic targets for OC.

## Materials and methods

### Patients’ samples

42 pairs of OC tissues and matched adjacent normal tissues were obtained from The First People’s Hospital of Shangqiu. The project was supported by the Ethics Committee of the First People’s Hospital of Shangqiu and informed consents were signed by all participants. All patients received not any preoperative treatment, the characteristics of clinical samples are enumerated in Table 1. Tumor stage was defined based on the Federation of Gynecology and Obstetrics (FIGO) guidelines [14]. Tumor-node-metastasis (TNM) stage was defined by the American Joint Committee on Cancer [15]. The isolated tissues were quickly placed in liquid nitrogen and preserved at − 80 °C ultra low-temperature refrigerator for subsequent experiments.

**Table 1:** The correlations of MIR4435-2HG with clinicopathological features of ovarian cancer *P < 0.05

Factor	No.	LNCRNA MIR4435-2HG n (%)		*P* value
		Low expression (n = 21)	High expression (n = 21)	
Age				0.682
≤ 55	22	10 (45.5)	12 (54.5)	
> 55	20	11 (55.0)	9 (45.0)	
Tumor size				0.031*
≤ 5	24	14 (58.3)	10 (41.7)	
> 5	18	7 (36.8)	11 (63.2)	
FIGO stage				0.026*
I + II	16	10 (62.5)	6 (37.5)	
III + IV	26	11 (42.3)	15 (57.3)	
Histological type				0.450
Serous	28	13 (46.4)	15 (53.6)	
Nonserous	14	8 (47.9)	6 (52.1)	
Lymph node metastasis				0.012*
No	23	15 (65.2)	8 (34.8)	
Yes	19	6 (31.6)	13 (68.4)	

*Statistically significant

### Cell culture and transfection

OC cell lines (SKOV3, Caov-3, A2780, and OVCAR3), normal ovarian cell line (ISOE80) and HEK293T cells were bought from Shanghai Institute of Biochemistry and Cellular Biology (Shanghai, China) and cultured with Roswell Park Memorial Institute (RPMI) 1640 medium (Invitrogen, Carlsbad, CA, USA) or Dulbecco’s modified Eagle’s medium (DMEM, Invitrogen) including 10% fetal bovine serum (FBS, Invitrogen) and 1% penicillin/streptomycin (Invitrogen) in a 5% CO2 incubator at 37 °C.

The sequences of MIR4435-2HG, miR-128-3p and CDK14 were searched from National Center for Biotechnology Information (NCBI). Small interfering RNA (siRNA) targeting MIR4435-2HG (si-MIR4435-2HG) and negative control (si-NC), miR-128-3p mimics/NC mimics, miR-128-3p inhibitor/NC inhibitor, pcDNA-MIR4435-2HG or CDK14 overexpression vector (MIR4435-2HG or CDK14)/pcDNA empty vector (Vector), lentiviral vector containing short hairpin RNA against MIR4435-2HG (sh-MIR4435-2HG) and negative control (sh-NC) were all constructed by Shanghai Sangon Biotech Co., Ltd. (Shanghai, China). Next, Lipofectamine 3000 (Invitrogen) was applied to perform cell transfection according to the manufacturer’s directions. After transfection for 48 h, the transfection efficacy was measured by quantitative real-time polymerase chain reaction (qRT-PCR).

### Chromogenic in situ hybridization (CISH)

CISH was carried out using the ISH Optimization Kit (FFPE) (Exiqon, Vedbaek, Denmark) in line with the product’s protocol. A biotin-labeled probe was used for MIR4435-2HG detection in OC tissue microarray (TMA) (Outdo Biotech, Shanghai, China), which consisted of 42 pairs of OC tissues and matched adjacent normal tissues. A digoxin-labeled probe of scrambled RNA served as the negative control (Exiqon) and a digoxin-labeled beta-actin probe was used as the positive control (Exiqon).

### qRT-PCR analysis

The total RNA from tissues and cell lines after transfection was extracted by TRIzol reagent (Invitrogen). A PrimeScript RT kit (Takara, Dalian, China) was used to quantify and synthesize complementary DNA (cDNA). Subsequently, the qRT-PCR reaction was performed according to the illustration of SYBR^®^ Premix ExTaq kit (TaKaRa) in ABI7500 instrument (Applied Biosystems Company, Oyster Bay, NY, USA). The primer sequences used in the study were as follows: MIR4435-2HG forward, 5′-GACTCTCCTACTGGTGCTTGGT-3′, and reverse, 5′-CACTGCCTGGTGAGCCTGTT-3′; miR-128-3p forward, 5′-TGCGGCAGTGGTTTTACCCTATG-3′, and reverse, 5′-CCAGTGCAGGGTCCGAGGT-3′; CDK14 forward, 5′-TGTCAGTACATGGACAAGCACCCT-3′, and reverse, 5′-TGTAAGACAGACCTCGCAGCAACT-3′; GAPDH forward, 5′-GCAAGAGCACAAGAGGAAG-3′, and reverse, 5′-TCTACATGGCAACTGTGAGG-3′; U6 forward, 5′-TGCGGGTGCTCGCTTCGGCAGC-3′, and reverse, 5′-CCAGTGCAGGGTCCGAGGT-3′. Glyceraldehyde-3-phosphate dehydrogenase (GAPDH) and U6 were acted as internal references. All data were calculated using the 2^−∆∆Ct^ method.

### MTT assay

Transfected SKOV3 and OVCAR3 (1 × 10^4^ cells/well) cells were inoculated into 96-well plates. Afterwards, culture medium with 10% MTT solution was added into each well. After incubation for 4 h, 100 μL detergent reagent was placed into 96-well plates and incubated devoid of light at room temperature for 2 h. The absorbance at 570 nm in each well at 24 h, 48 h, or 72 h was recorded under a microplate reader (Bio-Rad, Philadelphia, PA, USA).

### Flow cytometry assay

A PI/Annexin V Kit (Sigma, Saint Louis, MO, USA) was used to examine cell apoptosis rate. SKOV3 and OVCAR3 cells in exponential phase were collected and washed twice with PBS, then 5 × 10^5^ cells were resuspended in 100 μL binding buffer, and incubated with propidium iodide (PI) and Annexin V for 10 min in the dark at 37 °C, the apoptotic rate was observed by FACScan flow cytometer (Beckman Coulter, Inc. San Jose, CA, USA).

### Transwell assay

Migration assay was completed in 24-well transwell chambers (Millipore, Bedford, MA, USA). Transfected cells were cultured in the upper chambers without matrigel (Millipore) and 600 μL medium containing 10% FBS was put into the lower chambers. After 24 h, cells transferred to the surface of the lower chamber were fixed with 4% paraformaldehyde and stained with 0.1% crystal violet. Finally, the migratory cells were observed by a microscope (Olympus, Tokyo, Japan). Transwell invasion experiments are carried out by the same method with the upper chambers pre-coated with matrigel.

### Wound healing assay

SKOV3 and OVCAR3 cells were seeded in 6-well plates (5 × 10^5^ cells/well) and exposed to mitomycin C (Sigma) for 2 h. The cell monolayer was wounded with a pipette tip to create a scratch. Then, the cells were washed with PBS and the serum-free medium was added. Images were captured every 6 h following the initial scratch to evaluate cell migration rate.

### Western blot

Total proteins from tissues and cells were extracted by Radioimmunoprecipitation assay (RIPA) kit (Beyotime, Beijing, China) and the bicinchoninic acid (BCA) kit (Beyotime) was applied to evaluate the protein concentration. The protein was divided by polyacrylamide gel electrophoresis and transferred to specific membranes (Millipore). Subsequently, the membranes were maintained with 5% skimmed milk for 1 h and incubated overnight at 4 °C with the subsequent rabbit antibodies (Abcam, Cambridge, MA, USA), CDK14 (1:1000, Abcam), Bcl-2 (1:1000), Cleaved PARP (1:1000, Abcam), E-cadherin (1:1000, Abcam), Vimentin (1:1000, Abcam). GAPDH (1:1000, Abcam) was used as an internal control. Then, the membranes were maintained with the secondary antibody at 37 °C for 1 h. In the end, the levels of the visible protein were observed by the chemiluminescence detection system.

### In vivo experiments

BALB/c nude mice (n = 6, 4-week-old, female) were purchased from HFK bioscience Co., LTD (Beijing, China). SKOV3 cells were stably transfected with sh-MIR4435-2HG or sh-NC. Then, stably transfected SKOV3 cells were subcutaneously inoculated into the right flank of mice back. Five days after inoculation, the tumor volume was calculated every five days with a vernier caliper according to the formula: length × width^2^ × 0.5. After 30 days, all tumor tissues were excised and used for weighting and expression analysis. All animal procedures were approved by the Animal Care and Use Committee of The First People’s Hospital of Shangqiu.

### Dual-luciferase reporter assay

Starbase v2.0 (http://starbase.sysu.edu.cn/) was applied to predict the target miRNAs of MIR4435-2HG and molecular targets of miR-128-3p. The wild type (MIR4435-2HG WT) or mutant type (MIR4435-2HG MUT) containing the putative binding sites of miR-128-3p were amplified and cloned into the pGL4-control luciferase reporter vectors (Promega Corporation, Madison, WI, USA). Target sequence and mutation sequence were constructed according to potential binding sites of miR-128-3p on CDK14 3′UTR (CDK14 3′UTR WT and CDK14 3′UTR MUT). HEK293T cells were co-transfected with the reporter plasmid and miR-128-3p mimics or miR-NC by Lipofectamine 3000 (Invitrogen). After transfection for 48 h, the luciferase activity was analyzed by a dual-luciferase reporter assay system (Promega Corporation).

### RNA immunoprecipitation (RIP) assay

RIP assay was performed using the Magna RIP RNA-Binding Protein Immunoprecipitation Kit (Millipore) in agreement with the manufacturer’s instruction. SKOV3 and OVCAR3 cell lysates were obtained and incubated with RIP buffer containing magnetic beads conjugated with human anti-Argonaute2 (anti-Ago2) antibody (Millipore) or normal mouse IgG (control; Millipore). RNA was extracted from immunoprecipitate and analyzed by qRT-PCR.

### Pull-down assay

Biotinylated miR-128-3p or NC mimics were transfected into SKOV3 and OVCAR3 cells, respectively. The active cells were collected after transfection and treated with lysis buffer (Ambion, Austin, Texas, USA). Following the protocol of manufacturer, the cell lysate was incubated with Dynabeads M-280 Streptavidin (Invitrogen). Beads were rinsed and incubated with biotinylated miR-128-3p at 4 °C overnight. Then, the RNase-free lysis buffer was added into the beads with the immobilized miR-128-3p fragment and incubated for 1 h at 37 °C, then interacted RNAs were purified and evaluated by qRT-PCR.

### Statistical analysis

SPSS 22.0 (IBM Corp, Armonk, NY, USA) was used to complete the statistical analyses. The experimental data were displayed as mean ± standard deviation. The *t* test and one-way analysis of variance were adopted to analyze experimental data. The survival rate was evaluated with Kaplan–Meier. The difference was statistical significance when *P* < 0.05.

## Results

### MIR4435-2HG was highly expressed in OC tissues and cell lines

**Fig. 1:**
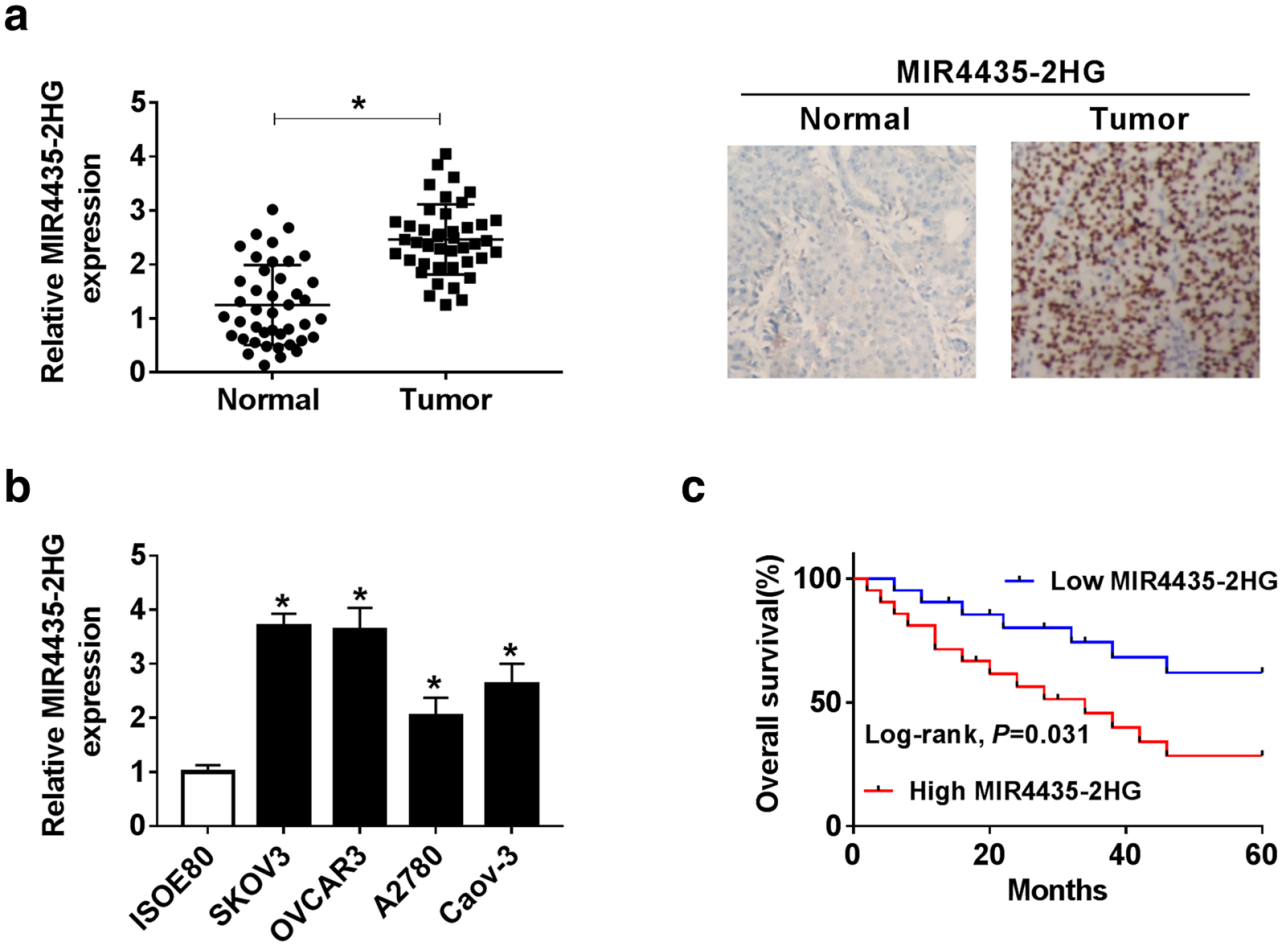
MIR4435-2HG was upregulated in OC tissues and cell lines. **a** The expression level of MIR4435-2HG in clinical OC tissues (n = 42) and normal tissues (n = 42) was detected by qRT-PCR. The abundance of MIR4435-2HG in tumor tissues and normal tissues was investigated by CISH. **b** The level of MIR4435-2HG in cultured cell lines was examined using qRT-PCR. **c** The correlation between MIR4435-2HG expression level and the overall survival of OC patients was analyzed by the Kaplan–Meier plot and log-rank test. *P < 0.05

The expression of MIR4435-2HG was examined in OC tissues and adjacent normal tissues. The results showed that the expression of MIR4435-2HG in OC tissues (n = 42) was significantly increased by 1.97 folds on average compared with adjacent normal tissues (n = 42) (*P* < 0.05) (Fig. 1a). Besides, CISH assay revealed that there was strong staining in tumor tissues but not in adjacent normal tissues, suggesting the high abundance of MIR4435-2HG in OC tissues (Fig. 1a). Compared with normal ovarian cell line ISOE80, the expression of MIR4435-2HG in OC cell lines (SKOV3, Caov-3, A2780, and OVCAR3) was notably increased, while SKOV3 and OVCAR3 cell lines represented with the higher MIR4435-2HG expression (*P* < 0.05, Fig. 1b). Subsequently, the prognostic values of MIR4435-2HG expression were analyzed by Kaplan–Meier, and it was shown that the survival time of patients with low MIR4435-2HG expression was significantly higher than those with high MIR4435-2HG expression (*P* < 0.05, Fig. 1c). Meanwhile, to explore the clinical significance of MIR4435-2HG in OC, the relationship between its expression pattern and clinicopathological characteristics was analyzed, and the data implied that the expression level of MIR4435-2HG was closely correlated with tumor size, FIGO stage and the lymph distant metastasis (*P* < 0.05, Table 1). These results demonstrated that high MIR4435-2HG expression was associated with poor prognosis.

### Knockdown of MIR4435-2HG inhibited malignant behaviors of OC cells

**Fig. 2:**
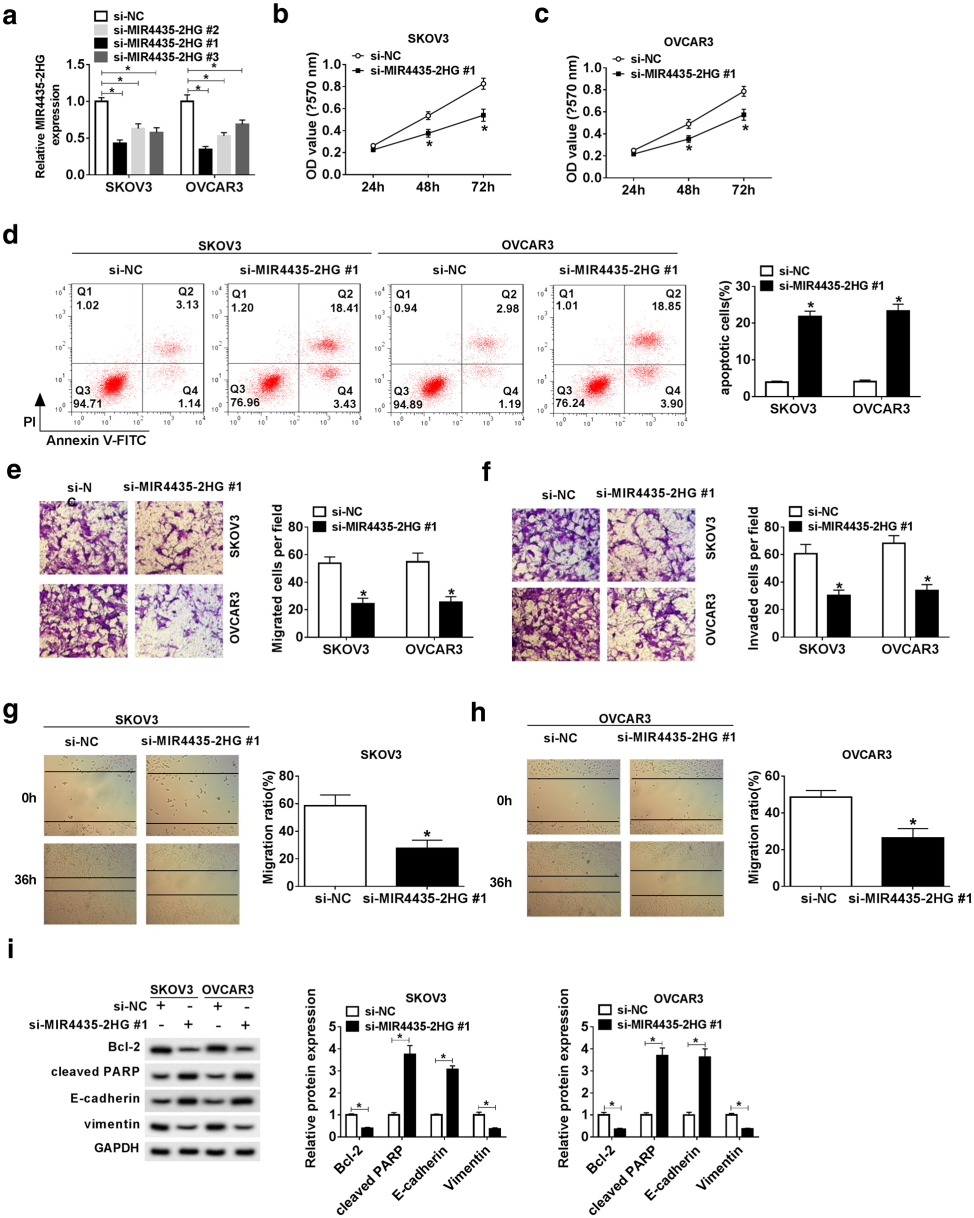
MIR4435-2HG knockdown inhibited the progression of OC cells. **a** The knockdown efficiency of MIR4435-2HG was verified using qRT-PCR. **b**, **c** Cell proliferation of SKOV3 and OVCAR3 cells with MIR4435-2HG knockdown in vitro was determined by MTT assay. **d** Cell apoptosis of SKOV3 and OVCAR3 cells after MIR4435-2HG knockdown was detected by flow cytometry assay. Q1: necrotic cells (AnnexinV-FITC)-/PI + ; Q2: late apoptotic or necrotic cells (AnnexinV + FITC) +/PI + ; Q3: early apoptotic cells (AnnexinV-FITC) +/PI-; Q4: unstained viable cells (AnnexinV-FITC)-/PI-. **e**, **f** The migration and invasion of SKOV3 and OVCAR3 cells after MIR4435-2HG knockdown were examined using transwell assay. **g**, **h** The wound healing assay was performed to observe the migration ratio in SKOV3 and OVCAR3 with MIR4435-2HG knockdown. **i** The protein levels of EMT markers (E-cadherin, Vimentin) and apoptosis indicators (Cleaved PARP, Bcl-2) in SKOV3 and OVCAR3 cells after MIR4435-2HG knockdown were analyzed by western blot. *P < 0.05

To examine the biological functions of MIR4435-2HG in OC cells, the expression of MIR4435-2HG was prevented by si-MIR4435-2HG in SKOV3 and OVCAR3 cells. The lowest MIR4435-2HG expression was caused by si-MIR4435-2HG #1 (0.42 folds on average), therefore si-MIR4435-2HG #1 was chosen for subsequent experimentations (*P* < 0.05, Fig. 2a). By performing MTT assay, MIR4435-2HG knockdown was shown to significantly retard the proliferative capacity of SKOV3 and OVCAR3 cells compared with the NC group (*P* < 0.05, Fig. 2b, c). The flow cytometry results showed that SKOV3 and OVCAR3 cells transfected with si-MIR4435-2HG induced apoptosis augment (*P* < 0.05, Fig. 2d). In transwell assay, the migration and invasion of SKOV3 and OVCAR3 cells were inhibited in the si-MIR4435-2HG group compared with the si-NC group (*P* < 0.05, Fig. 2e and f). Besides, the wound healing assay presented that MIR4435-2HG knockdown suppressed the migration rate of SKOV3 and OVCAR3 cells compared with NC group (Fig. 2g and h). Moreover, the protein expression of Cleaved PARP and E-cadherin was activated by MIR4435-2HG knockdown, while Bcl-2 and Vimentin were restricted (*P* < 0.05, Fig. 2i). All the data indicated that depletion of MIR4435-2HG promoted apoptosis pathway but inhibited Epithelial-to-mesenchymal transition (EMT) progression, and MIR4435-2HG knockdown can act as a tumor inhibitor in the development of OC.

### MIR4435-2HG knockdown inhibited tumor growth in vivo

**Fig. 3:**
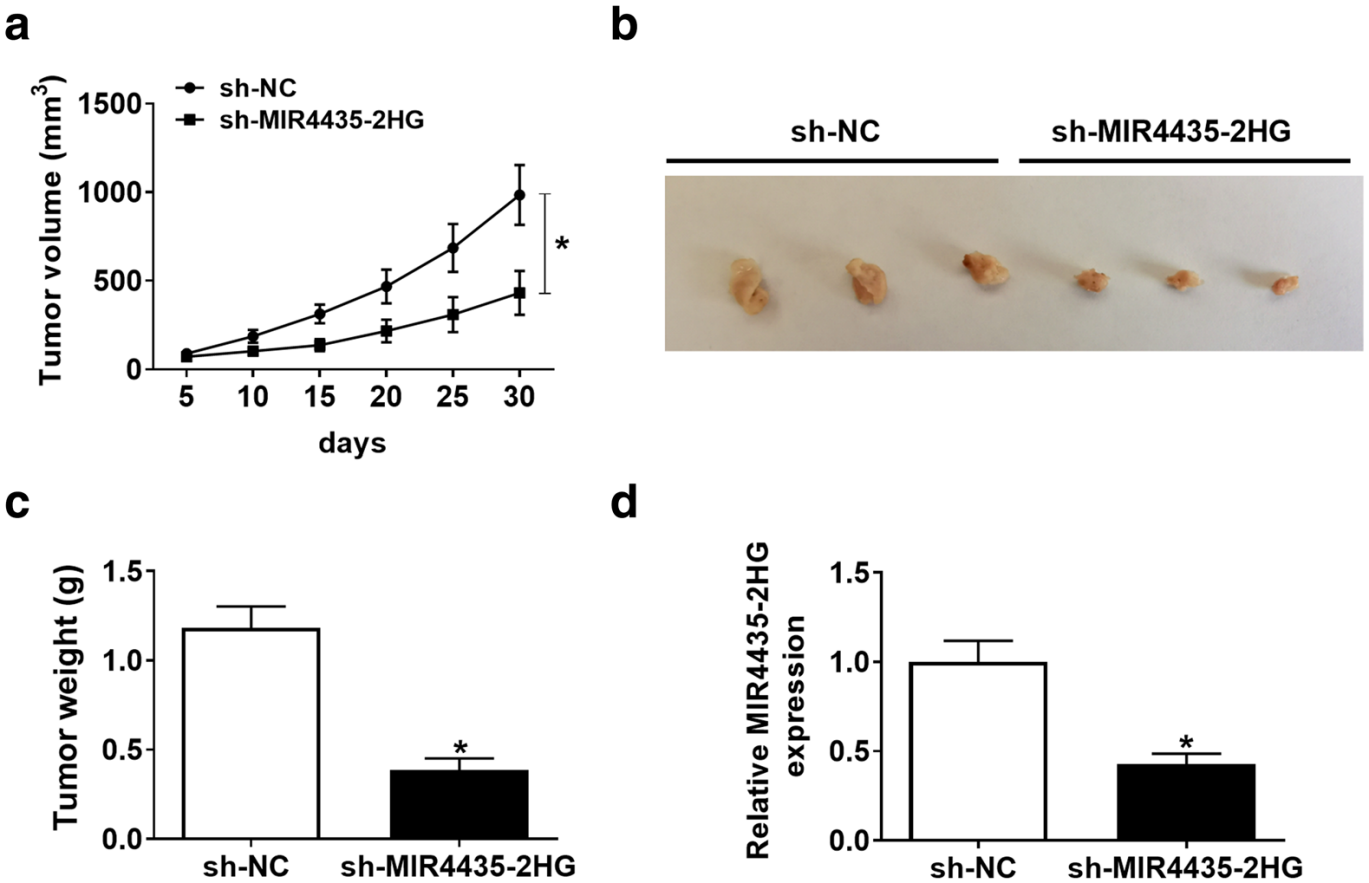
MIR4435-2HG knockdown inhibited tumor growth in vivo. SKOV3 cells transfected with sh-MIR4435-2HG or sh-NC were injected into the nude mice. **a** The tumor volume was calculated every 5 days. **b** The size of excised tumor tissues. **c** The weight of excised tumor tissues. **d** The expression of MIR4435-2HG in excised tumor tissues. *P < 0.05

To ascertain the role of MIR4435-2HG in vivo, the sh-MIR4435-2HG transfected SKOV3 cells were subcutaneously injected into the mice. Compared with sh-NC injection, the tumor volume and tumor weight were strikingly reduced in the sh-MIR4435-2HG groups (Fig. 3a–c). Then, the expression of MIR4435-2HG in excised tumor tissues was checked, and the result showed that the level of MIR4435-2HG was significantly decreased by 0.428 folds on average in the sh-MIR4435-2HG group relative to the sh-NC group (Fig. 3d). These data demonstrated that MIR4435-2HG knockdown suppressed tumor growth in vivo.

### MIR4435-2HG targeted miR-128-3p as a sponge

**Fig. 4:**
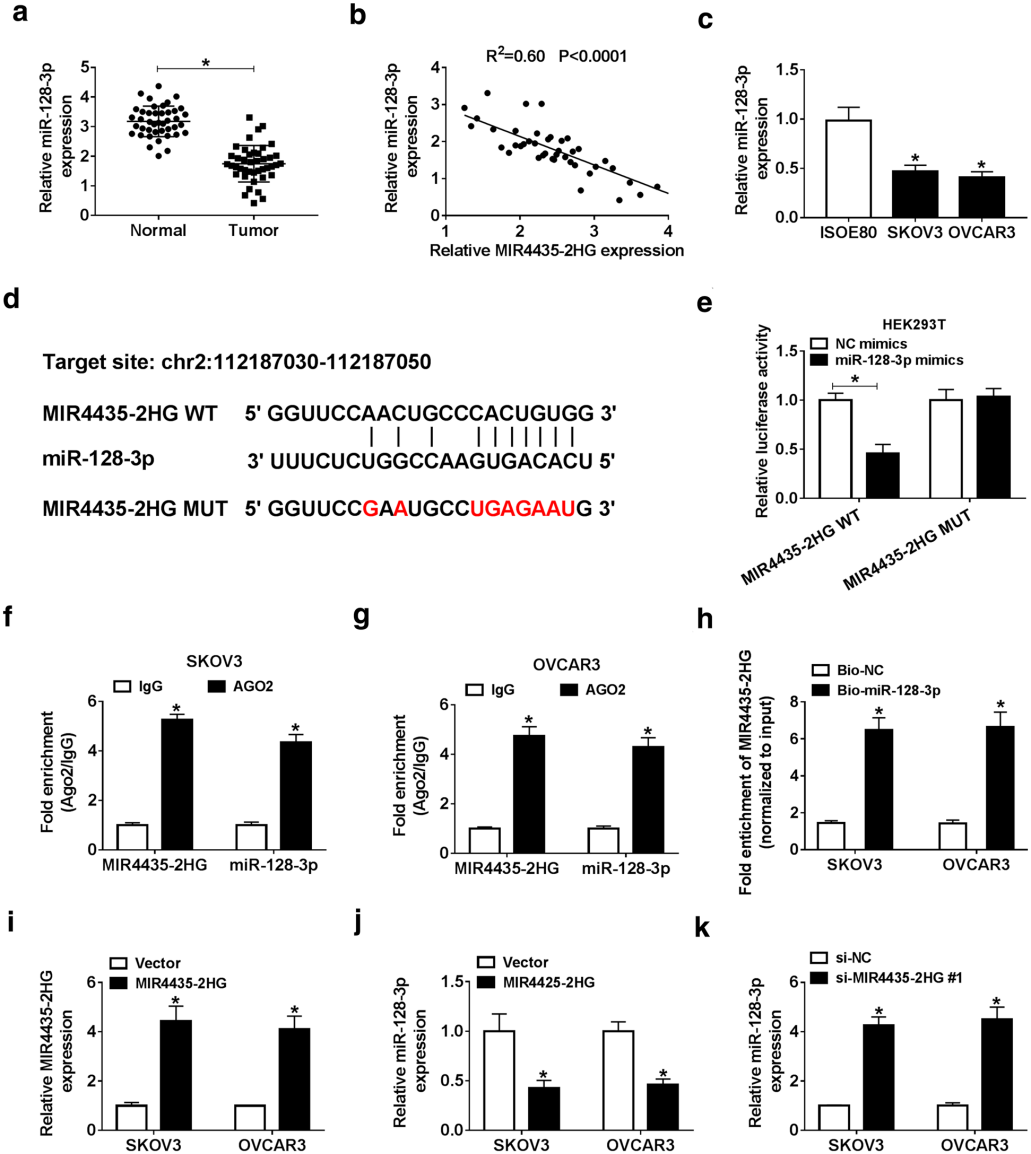
MIR4435-2HG targeted miR-128-3p to regulate its expression. **a** The expression of miR-128-3p in OC tissues (n = 42) and adjacent noncancerous tissues (n = 42) was measured by qRT-PCR. **b** The correlation between MIR4435-2HG expression and miR-128-3p expression in OC tissues was analyzed by Spearman’s correlation analysis. **c** The expression of miR-128-3p in ISOE80, SKOV3, and OVCAR3 cells was determined by qRT-PCR. **d** Sequences of miR-128-3p with the putative binding sites of MIR4435-2HG were predicted by starBase v2.0. **e** The relationship between miR-128-3p and MIR4435-2HG was verified using dual-luciferase reporter assay. **f**–**h** The interaction between MIR4435-2HG and miR-128-3p in SKOV3 and OVCAR3 cells was further verified by RIP assay and pull-down assay. **i**, **j** qRT-PCR was used to manifest the effect of MIR4435-2HG upregulation on the expression levels of MIR4435-2HG and miR-128-3p in SKOV3 and OVCAR3 cells. **k** The expression of miR-128-3p in SKOV3 and OVCAR3 cells transfected with si-MIR4435-2HG was examined by qRT-PCR. *P < 0.05

We examined the expression of miR-128-3p in 42 paired OC samples and normal tissues from the patients by qRT-PCR, and the data showed that miR-128-3p was reduced in OC tissues (*P* < 0.05, Fig. 4a). Besides, the expression of miR-128-3p was negatively associated with the MIR4435-2HG expression in OC tissues (R^2^ = 0.60, P < 0.0001, Fig. 4b). Similarly, miR-128-3p was also notably weakened in SKOV3 (0.48 folds on average) and OVCAR3 (0.42 folds on average) cells compared with that in normal cells (ISOE80) (*P* < 0.05, Fig. 4c). The complementary binding sites between miR-128-3p and MIR4435-2HG were predicted by StarBase v2.0 (Fig. 4d). The dual-luciferase reporter assay showed that the luciferase activity in HEK293T cells with MIR4435-2HG-WT and miR-128-3p mimics cotransfection was notably decreased by 0.46 folds on average relative to NC mimics, while the luciferase activity had no noticeable change in MIR4435-2HG-MUT group (*P* < 0.05, Fig. 4e). RIP assay exhibited that MIR4435-2HG and miR-128-3p were notably enriched in the AG02 RIP but not IgG RIP (*P* < 0.05, Fig. 4f and g). RNA pull-down assay illustrated that MIR4435-2HG-MUT was obviously enriched in the pellet pulled down by miR-128-3p compared with NC (*P* < 0.05, Fig. 4h). MIR4435-2HG overexpression could upregulate MIR4435-2HG and decrease the expression of miR-128-3p (*P* < 0.05, Fig. 4i and j). The expression of miR-128-3p could be increased by MIR4435-2HG knockdown (*P* < 0.05, Fig. 4k). The results indicated that MIR4435-2HG acted as a miRNA sponge and negatively regulated the expression of miR-128-3p.

### MIR4435-2HG abolished the effects of miR-128-3p overexpression on the progression of OC cells

**Fig. 5:**
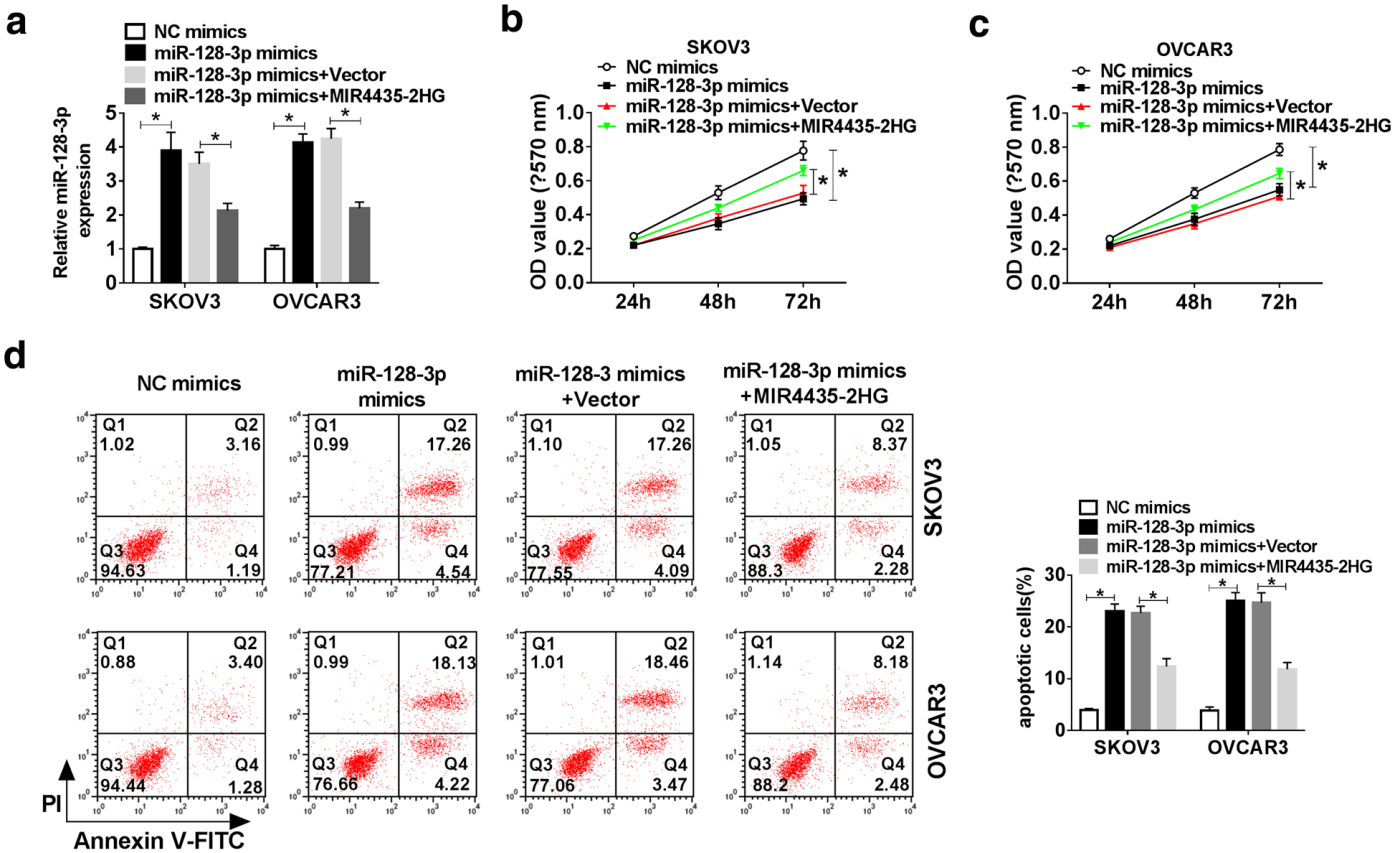
MIR4435-2HG overexpression reversed the effects of miR-128-3p upregulation on cell proliferation and apoptosis in OC cells. **a** The expression of miR-128-3p in SKOV3 and OVCAR3 cells transfected with miR-128-3p mimics, NC mimics, miR-128-3p mimics + MIR4435-2HG or miR-128-3p mimics + Vector was detected by qRT-PCR. **b**, **c** Cell proliferation was detected using MTT assay. **d** The apoptosis of SKOV3 and OVCAR3 cells was measured by flow cytometry. Q1: necrotic cells (AnnexinV-FITC)-/PI+; Q2: late apoptotic or necrotic cells (AnnexinV + FITC) +/PI+; Q3: early apoptotic cells (AnnexinV-FITC) +/PI−; Q4: unstained viable cells (AnnexinV-FITC)-/PI−. *P < 0.05

**Fig. 6:**
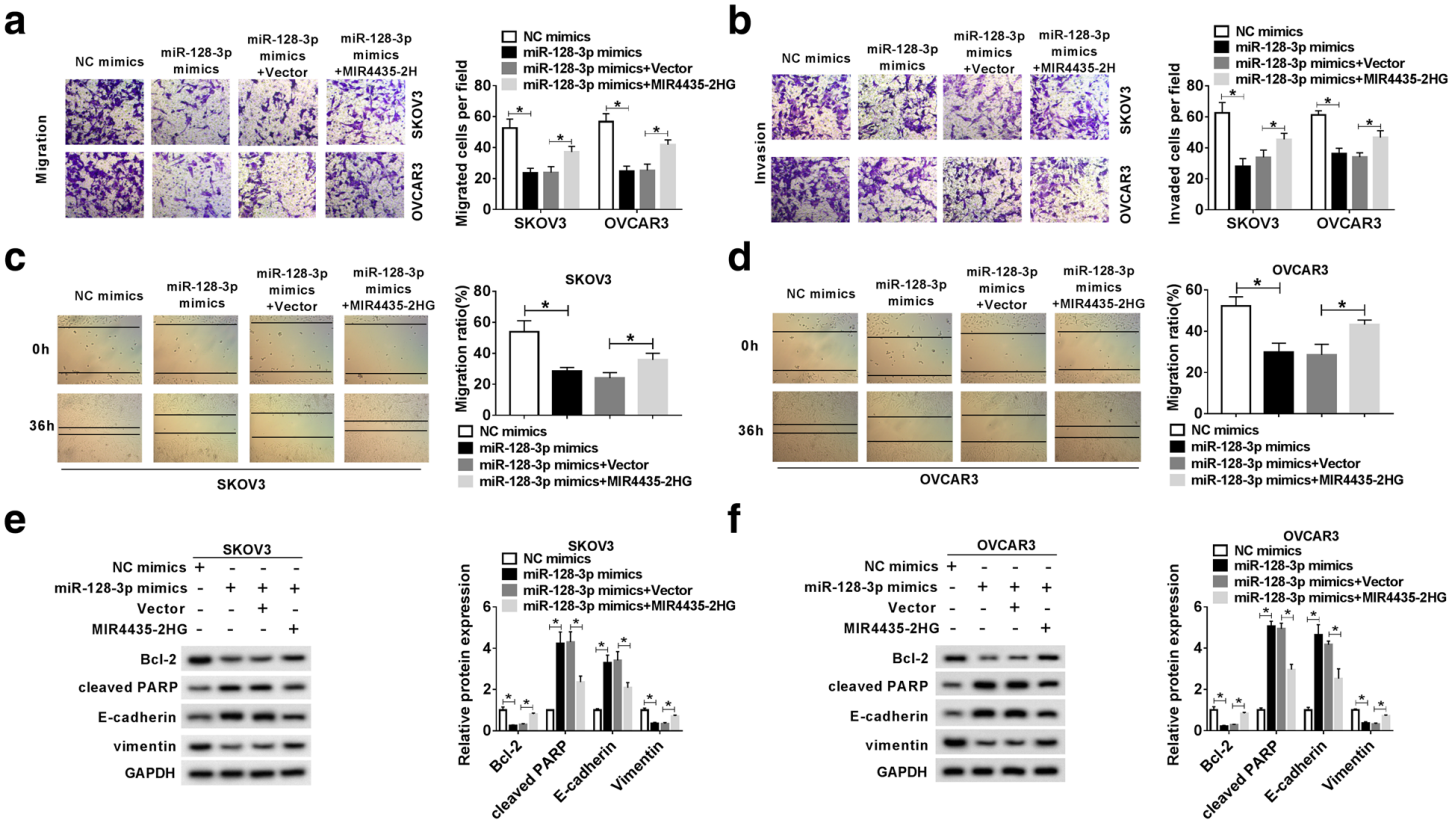
MIR4435-2HG upregulation blocked the effects of miR-128-3p upregulation on cell migration and invasion in OC cells. **a**, **b** Transwell assay was used to detect the invasion and migration of SKOV3 and OVCAR3 cells. **c**, **d** The migration ration was observed by wound healing assay. **e**, **f** The levels of E-cadherin, Vimentin, Cleaved PARP and Bcl-2 were analyzed by western blot. *P < 0.05

To explore whether MIR4435-2HG targeted miR-128-3p to inhibit the biological function of miR-128-3p, the rescue experiments were performed. We firstly examined the level of miR-128-3p in SKOV3 and OVCAR3 cells, and the results showed that miR-128-3p was reinforced by the transfection of miR-128-3p mimics but impaired by the transfection of miR-128-3p mimics + MIR4435-2HG (*P* < 0.05, Fig. 5a). MTT assay showed that cell viability in the miR-128-3p mimics group was decreased, while it was recovered in the miR-128-3p mimics + MIR4435-2HG group (*P* < 0.05, Fig. 5b and c). Moreover, miR-128-3p mimics significantly induced apoptosis in both SKOV3 and OVCAR3 cells compared with NC mimics, while MIR4435-2HG upregulation led to opposite effects (*P* < 0.05, Fig. 5d). Besides, miR-128-3p mimics could significantly suppress the migration and invasion abilities of SKOV3 and OVCAR3 cells, which were restored by MIR4435-2HG overexpression (*P* < 0.05, Fig. 6a and b). The wound healing assay presented that the migration ratio of SKOV3 and OVCAR3 cells was blocked by miR-128-3p mimics transfection but regained by miR-128-3p mimics + MIR4435-2HG transfection (*P* < 0.05, Fig. 6c and d). MiR-128-3p mimics increased the levels of Cleaved PRAP and E-cadherin, and decreased the levels of Bcl-2 and Vimentin, while these consequences could be overturned by MIR4435-2HG upregulation in both SKOV3 and OVCAR3 cells (*P* < 0.05, Fig. 6e and f). Taken together, these data indicated that MIR4435-2HG sponged miR-128-3p to promote the progression of OC cells.

### CDK14 was a target mRNA of miR-128-3p

**Fig. 7:**
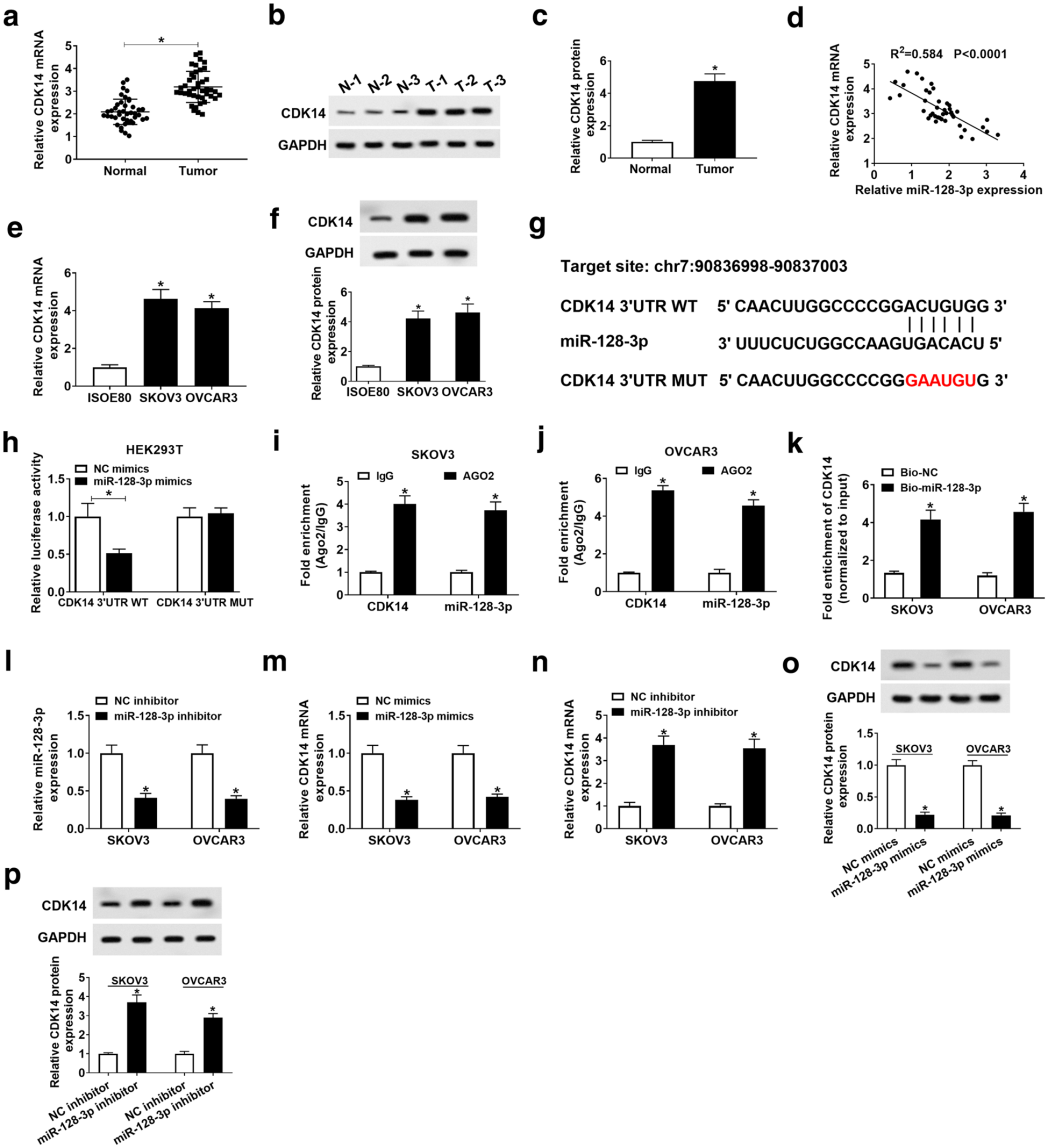
CDK14 is a target of miR-128-3p. **a**–**c** The expression of CDK14 in OC tissues (n = 42) and adjacent normal tissues (n = 42) was detected by qRT-PCR and western blot. **d** The correlation between CDK14 and miR-128-3p expression was analyzed by Spearman’s correlation analysis. **e**, **f** The expression level of CDK14 in ISOE80, SKOV3, and OVCAR3 cells was examined by qRT-PCR and western blot, respectively. **g** The specific binding regions between CDK14 and miR-128-3p were obtained from the online software starbase. **h**–**k** The relationship between CDK14 and miR-128-3p was confirmed by dual-luciferase reporter assay, RIP assay and RNA pull-down assay. **l** The expression of miR-128-3p in SKOV3 and OVCAR3 cells transfected with miR-128-3p inhibitor was analyzed by qRT-PCR. **m**–**p** The expression of CDK14 at mRNA and protein levels in SKOV3 and OVCAR3 cells transfected with miR-128-3p inhibitor or mimics was measured by qRT-PCR and western blot. *P < 0.05

The data from qRT-PCR and western blot elucidated that CDK14 expression was elevated in OC tissues (n = 42) relative to normal tissues (n = 42) (*P* < 0.05, Fig. 7a–c). Besides, the miR-128-3p expression was negatively related to the CDK14 expression in OC tissue samples (*P* < 0.05, Fig. 7d). Consistently, CDK14 expression was significantly upregulated in SKOV3 (4.62 folds on average) and OVCAR3 (4.13 folds on average) cells (*P* < 0.05, Fig. 7e and f). Next, the binding site between CDK14 3′UTR and miR-128-3p predicted by starBase v2.0 were mutated and used for dual-luciferase reporter assay (Fig. 7g). The data showed that miR-128-3p mimics and CDK14 3′UTR WT cotransfection caused the declined luciferase activity by 0.51 folds on average, while miR-128-3p mimics and CDK14 3′UTR MUT cotransfection did not alter the luciferase activity (*P* < 0.05, Fig. 7h). In addition, CDK14 and miR-128-3p could notably be loaded in the AGO2 RIP but not IgG RIP (*P* < 0.05, Fig. 7i and j). Moreover, CDK14 was richly expressed in the pellet pulled down by miR-128-3p, which confirmed the interaction between CDK14 and miR-128-3p (*P* < 0.05, Fig. 7k). MiR-128-3p was downregulated by miR-128-3p inhibitor in SKOV3 and OVCAR3 cells (*P* < 0.05, Fig. 7l). Overexpression of miR-128-3p could suppress CDK14 expression, while miR-128-3p inhibition could increase the expression of CDK14 (*P* < 0.05, Fig. 7m–p). These data suggested that CDK14 was a target of miR-128-3p.

### MiR-128-3p overexpression abrogated the role of CDK14 overexpression in OC cells

**Fig. 8:**
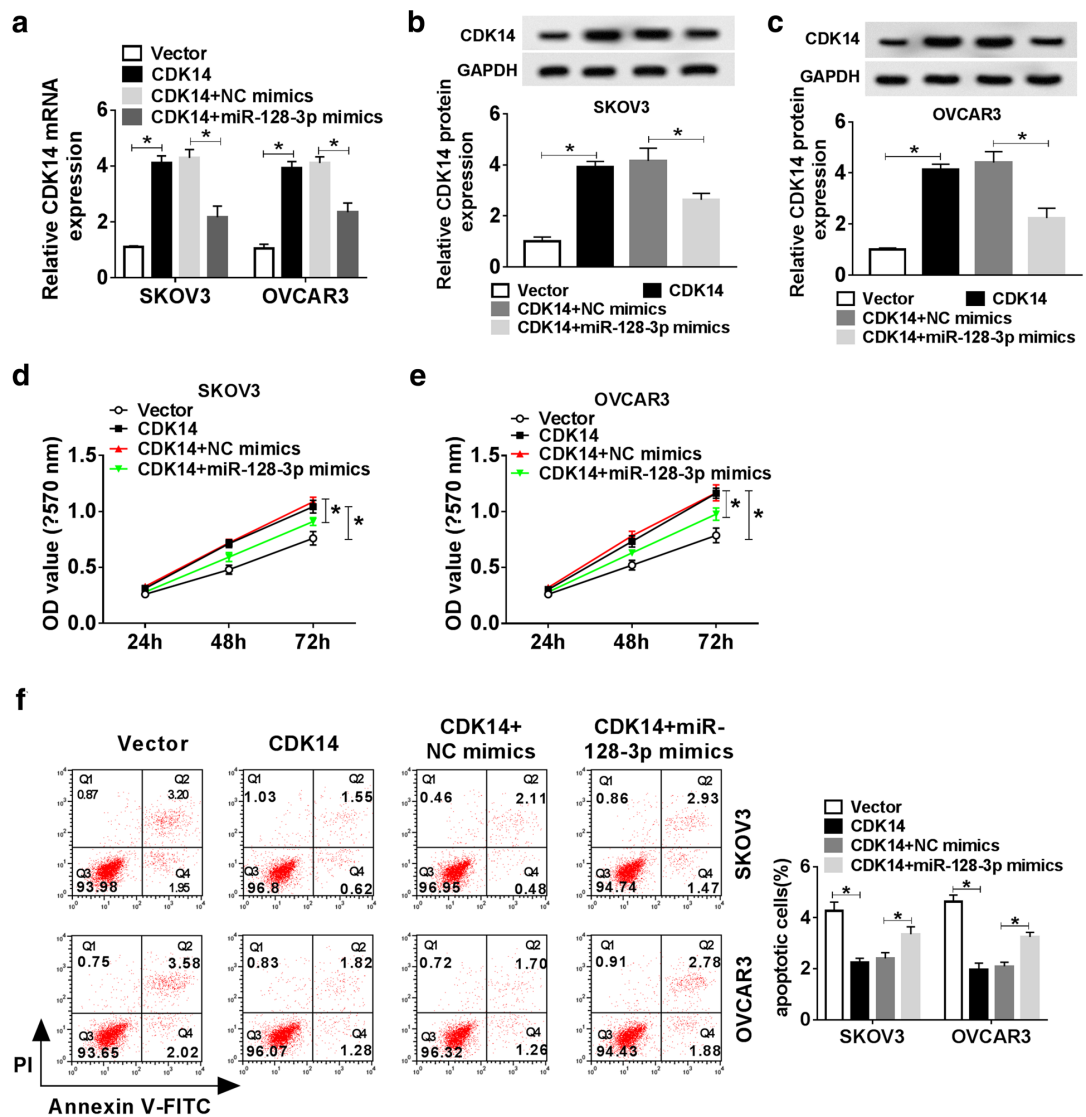
MiR-128-3p abolished the effects of CDK14 on cell proliferation and apoptosis in OC cells. SKOV3 and OVCAR3 were transfected with CDK14, Vector, CDK14 + miR-128-3p mimics or CDK14 + NC mimics. **a**–**c** The expression of CDK14 was evaluated by qRT-PCR and western blot analysis. **d**, **e** Cell proliferation was detected by MTT assay. **f** The apoptosis rate of SKOV3 and OVCAR3 cells was measured by flow cytometry. Q1: necrotic cells (AnnexinV-FITC)-/PI + ; Q2: late apoptotic or necrotic cells (AnnexinV + FITC) +/PI+; Q3: early apoptotic cells (AnnexinV-FITC) +/PI−; Q4: unstained viable cells (AnnexinV-FITC)-/PI−. *P < 0.05

**Fig. 9:**
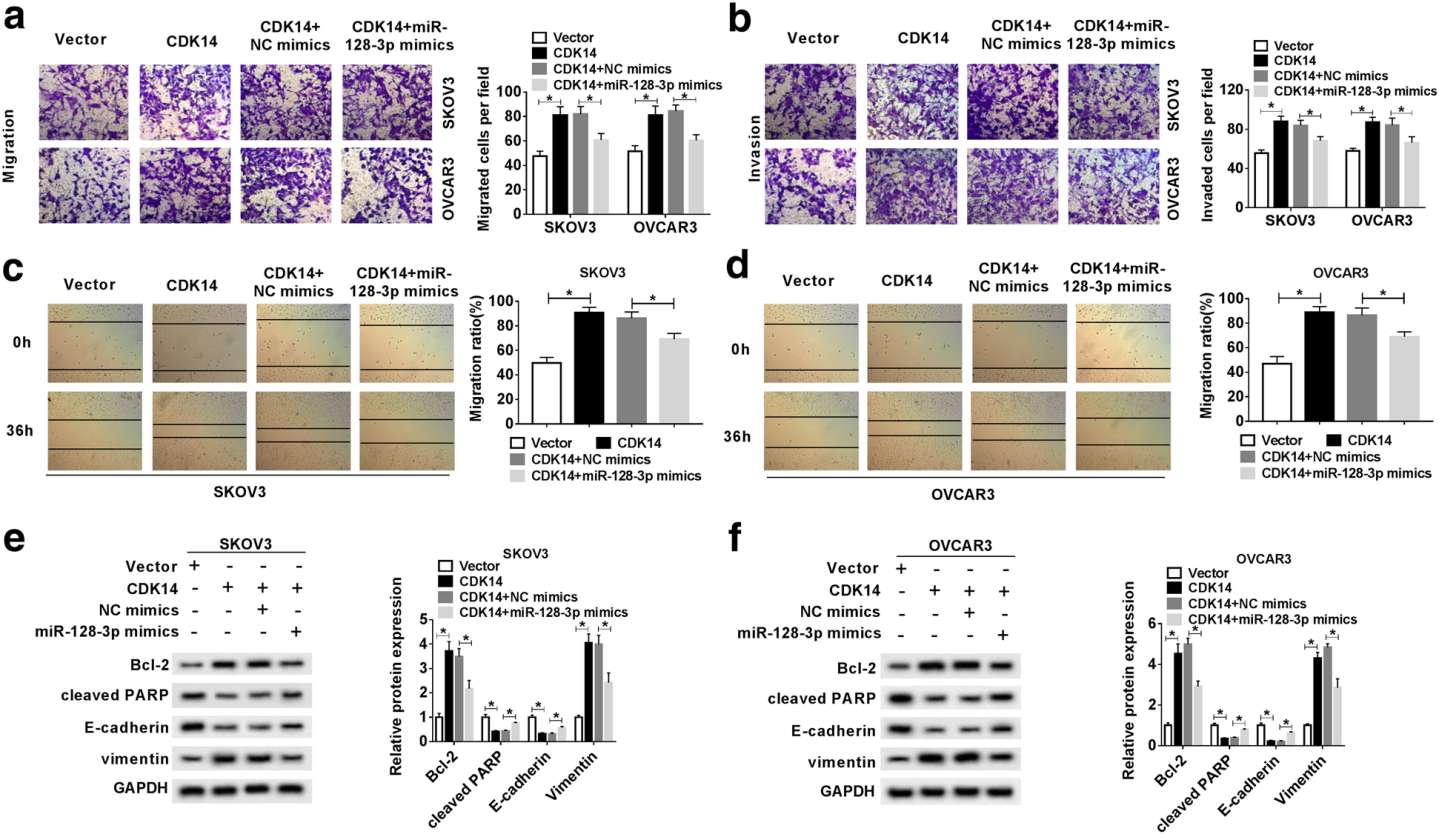
MiR-128-3p hindered the effects of CDK14 on cell migration and invasion in OC cells. **a**, **b** Cell migration and invasion in SKOV3 and OVCAR3 cells were determined by transwell assay. **c**, **d** The migration ratio was monitored according to the wound healing assay. **e**, **f** The expression of E-cadherin, Vimentin, Cleaved PARP and Bcl-2 in SKOV3 and OVCAR3 cells was measured by western blot. *P < 0.05

To investigate whether miR-128-3p interacted with CDK14 to inhibit the role of CDK14, SKOV3 and OVCAR3 cells were introduced with CDK14 or CDK14 + miR-128-3p mimics, Vector or CDK14 + NC mimics as the control. We noticed that the expression of CDK14 at both mRNA and protein levels elevated by CDK14 transfection was decreased in the CDK14 + miR-128-3p mimics group (*P* < 0.05, Fig. 8a–c). CDK14 overexpression could significantly enhance the proliferative capacity of SKOV3 and OVCAR3 cells, while the enhanced proliferation could be abolished by miR-128-3p mimics (*P* < 0.05, Fig. 8d and e). On the contrary, cell apoptosis was decreased by CDK14 overexpression but partially reversed by miR-128-3p mimics (*P* < 0.05, Fig. 8f). Next, we evaluated migration and invasion of SKOV3 and OVCAR3 cells using transwell assays. CDK14 transfection could significantly elevate the migration and invasion abilities of SKOV3 and OVCAR3 cells, while these enhanced abilities could be partially repressed by miR-128-3p mimics (*P* < 0.05, Fig. 9a and b). The wound healing assay revealed that CDK14 overexpression promoted the migration ratio, while miR-128-3p mimics partially restrained the role of CDK14 (*P* < 0.05, Fig. 9c and d). Additionally, the data from western blot assay depicted that CDK14 upregulation significantly increased the levels of Bcl-2 and Vimentin, whereas miR-128-3p overexpression reversed the positive effects of CDK14 on these proteins. Similarly, CDK14 generated a significant reduction in the levels of Cleaved PRAP and E-cadherin both in SKOV3 and OVCAR3 cells, which were augmented after the transfection of miR-128-3p mimics (*P* < 0.05, Fig. 9e and f). These analyses manifested that miR-128-3p bound to CDK14 and blocked the role of CDK14.

### Knockdown of MIR4435-2HG downregulated the expression of CDK4 via regulating the expression of miR-128-3p

**Fig. 10:**
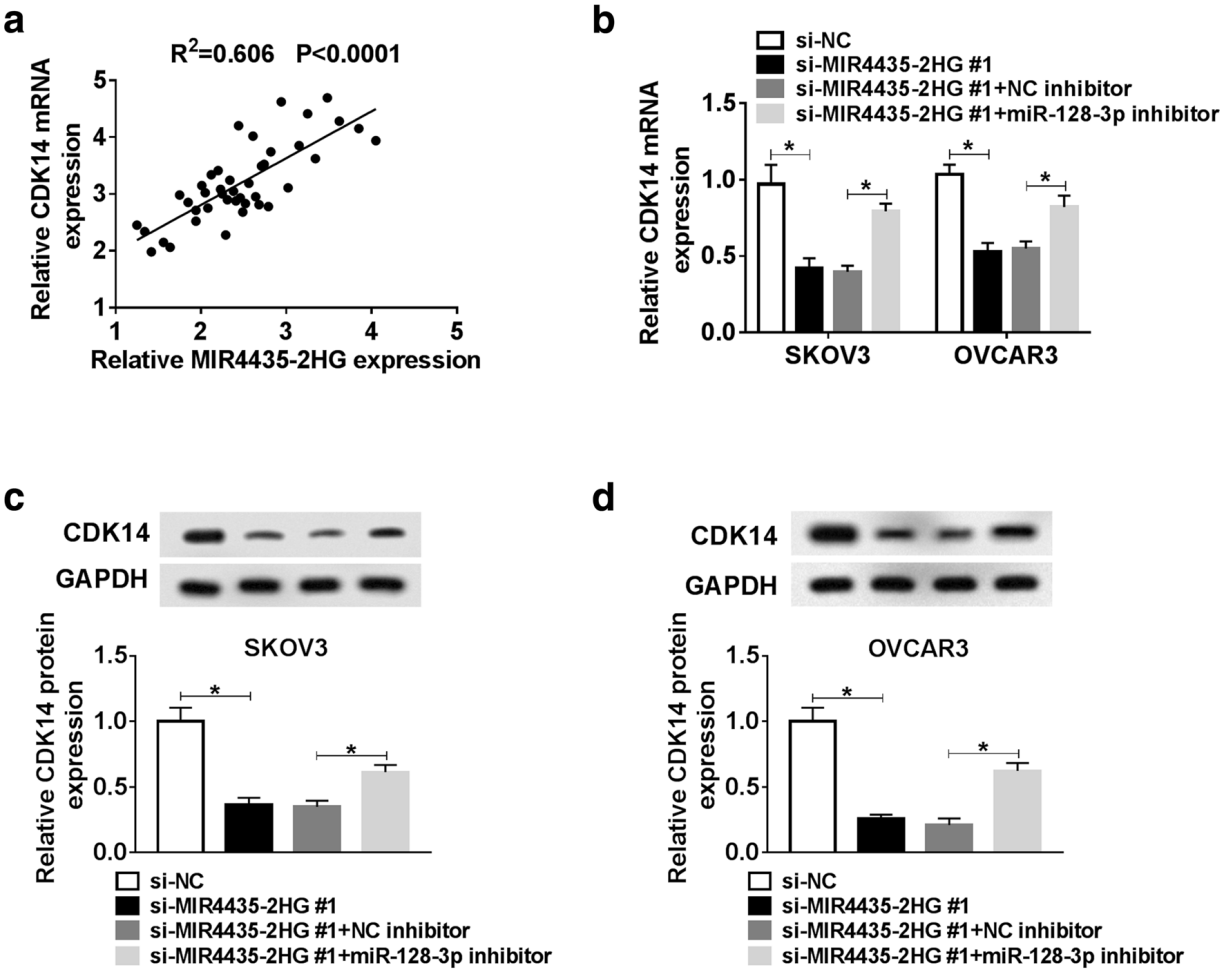
MIR4435-2HG knockdown downregulated CDK14 expression via sponging miR-128-3p in OC. **a** The correlation between CDK14 expression and MIR4435-2HG expression in OC tissues was analyzed by Spearman’s correlation analysis. **b**–**d** The mRNA and protein levels of CDK14 in SKOV3 and OVCAR3 cells transfected with si-MIR4435-2HG#1, si-NC, si-MIR4435-2HG#1 + miR-128-3p inhibitor or si-MIR4435-2HG#1 + NC inhibitor were examined using qRT-PCR and western blot. *P < 0.05

Further analysis revealed that CDK14 expression was positively correlated with the MIR4435-2HG expression in OC tissues (R^2^ = 0.606, *P* < 0.0001, Fig. 10a). The qRT-PCR analysis showed that CKD14 was reduced after the transfection of si-MIR4435-2HG#1 in SKOV3 and OVCAR3 cells, which was accelerated by miR-128-3p inhibitor (*P* < 0.05, Fig. 10b). We found that knockdown of MIR4435-2HG decreased the protein level of CDK14, but the effect was significantly reversed after the inhibition of miR-128-3p (*P* < 0.05, Fig. 10c and d). These data suggested that MIR4435-2HG knockdown depleted the expression of CDK14 by competitively binding to miR-128-3p in SKOV3 and OVCAR3 cells.

## Discussion

Accumulated research suggested that MIR4435-2HG played a critical role in multiple tumors, which can function as an oncogene to induce cancer cell reproduction and metastasis [9, 16]. Qian et al. found that over-expression of MIR4435-2HG contributed to lung cancer progression and was related to histological grade and lymph node metastasis [17]. Previous data showed that MIR4435-2HG could be regarded as a prognostic marker of OC [10]. However, the relationship between MIR4435-2HG and OC progression remains elusive. Thereby, we aimed to explore the specific role of MIR4435-2HG in OC.

In the study, qRT-PCR results suggested that MIR4435-2HG was greatly increased in both OC tissues and cell lines, and MIR4435-2HG overexpression was closely related to large tumor size, FIGO stage (III + IV), distant lymph metastasis, and poor survival in OC tissues. We also found that knockdown of MIR4435-2HG repressed proliferation, invasion, metastasis, and triggered apoptosis in SKOV3 and OVCAR3 cells. These results indicated that MIR4435-2HG might act as a carcinogen in OC, which was consistent with the previous study [10].

LncRNAs affected the occurrence and development of tumors via post-transcriptional regulation [18]. Multiple lncRNAs may regulate gene expression by isolating miRNAs, thus reducing the number of available miRNAs in cells. Therefore, lncRNA acts as a depressor of miRNA function and further serves as an activator of gene expression, which served as a competing endogenous RNA (ceRNA) to modulate the expression of the target gene via sponging miRNAs [19, 20]. In the current literatures, MIR4435-2HG was reported to exert ceRNA function in osteoarthritis [21], glioma [22], hepatocellular carcinoma [9] and lung cancer [17]. Similarly, we assumed that MIR4435-2HG functioned as an oncogene in OC via a ceRNA mode. To test this hypothesis, bioinformatic analysis of MIR4435-2HG-miRNA prediction was performed through online software, and the target binding was verified by a dual-luciferase reporter and pull-down assay. All the data indicated that miR-128-3p was a direct target of MIR4435-2HG. It was well known that miR-128-3p was an inhibitor in various tumors [23, 24]. Our data showed that the level of miR-128-3p was dramatically reduced in OC tissues and cell lines, which was inversely correlated with MIR4435-2HG expression in OC tissues. These results indicated that miR-128-3p was a tumor inhibitor in OC.

MiRNA acted as a tumor suppressor through the restraint of its target genes to participate in OC progression. We inferred CDK14 as a target of miR-128-3p containing the putative miRNA response sequences within its 3′-UTR by starBase v2.0, and the result indicated that CDK14 was a promising candidate gene of miR-128-3p. CDK14 was reported to be involved in the progression of various cancers, including OC. Jin et al. showed that high expression of SNHG15 facilitated NSCLC development through the acceleration of CDK14 and sponging miR-486 [25]. Li et al. pointed that overexpression of CDK14 reversed the depressant effects of miR-542-3p on OC cells [26], while CDK14 knockdown restrained cell proliferation, invasion and migration in OC [27]. Here, we found a remarkable negative correlation between the expression levels of CDK14 and miR-128-3p in OC tissues. CDK14 was a direct target of miR-128-3p in SKOV3 and OVCAR3 cells, which indicated that the ceRNA system existed among MIR4435-2HG, miR-128-3p and CDK14 in OC. Next, we wondered that whether MIR4435-2HG/miR-128-3p/CDK14 axis conduced to the progression of OC in vitro. The data presented that the effect of MIR4435-2HG knockdown reduced CDK14 expression level by the promotion of miR-128-3p.

## Conclusions

**Fig. 11:**
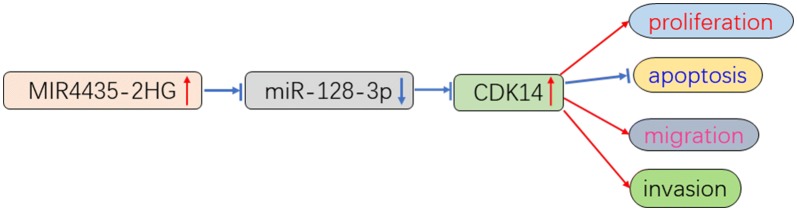
The MIR4435-2HG/miR-128-3p/CDK14 axis in OC progression

In summary, lncRNA MIR4435-2HG was regarded as a vital mediator of cell growth in OC. MIR4435-2HG was obviously upregulated in OC tissues and cell lines, and MIR4435-2HG knockdown repressed OC progression by acting as a ceRNA to downregulate CDK14 through competitively binding to miR-128-3p (Fig. 11). These results indicated that MIR4435-2HG/miR-128-3p/CDK14 axis may be a potential therapeutic basis for the treatment of OC.

## Data Availability

The analyzed data sets generated during the present study are available from the corresponding author on reasonable request.

## Abbreviations

OC: Ovarian cancer
qRT-PCR: Quantitative real time polymerase chain reaction
CDK14: Cyclin-dependent kinase 14
OS: Overall survival
WHO: World Health Organization
FBS: Fetal bovine serum
NCBI: National Center for Biotechnology Information
NC: Negative control
RIPA: Radioimmunoprecipitation assay
BCA: Bicinchoninic acid

## References

[1] Siegel RL (2019). Cancer statistics, 2019. CA Cancer J Clin.

[2] Cong J (2019). Therapeutic effect of bevacizumab combined with paclitaxel and carboplatin on recurrent ovarian cancer. J Buon.

[3] Jayson GC (2014). Ovarian cancer. Lancet.

[4] Reid BM (2017). Epidemiology of ovarian cancer: a review. Cancer Biol Med.

[5] Yarmishyn AA (2015). Long noncoding RNAs: a potential novel class of cancer biomarkers. Front Genet.

[6] Deng R (2016). High expression of the newly found long noncoding RNA Z38 promotes cell proliferation and oncogenic activity in breast cancer. J Cancer.

[7] Prensner JR (2011). The emergence of lncRNAs in cancer biology. Cancer Discov.

[8] Ouyang W (2019). LncRNA MIR4435-2HG predicts poor prognosis in patients with colorectal cancer. PeerJ.

[9] Kong Q (2019). The lncRNA MIR4435-2HG is upregulated in hepatocellular carcinoma and promotes cancer cell proliferation by upregulating miRNA-487a. Cell Mol Biol Lett.

[10] Gong J (2019). LncRNA MIR4435-2HG is a potential early diagnostic marker for ovarian carcinoma. Acta Biochim Biophys Sin.

[11] Zhao J (2019). MiR-128-3p suppresses breast cancer cellular progression via targeting LIMK1. Biomed Pharmacother.

[12] Huo L (2019). miR-128-3p inhibits glioma cell proliferation and differentiation by targeting NPTX1 through IRS-1/PI3K/AKT signaling pathway. Exp Ther Med.

[13] Ou-Yang J (2017). Cyclin-dependent kinase 14 promotes cell proliferation, migration and invasion in ovarian cancer by inhibiting Wnt signaling pathway. Gynecol Obstet Invest.

[14] Wright JD (2019). Prognostic performance of the 2018 International Federation of Gynecology and Obstetrics Cervical Cancer Staging Guidelines. Obstet Gynecol.

[15] Edge SB (2010). The American Joint Committee on Cancer: the 7th edition of the AJCC cancer staging manual and the future of TNM. Ann Surg Oncol.

[16] Reon BJ (2018). LINC00152 Promotes invasion through a 3′-hairpin structure and associates with prognosis in glioblastoma. Mol Cancer Res.

[17] Qian H (2018). The lncRNA MIR4435-2HG promotes lung cancer progression by activating beta-catenin signalling. J Mol Med.

[18] Dykes IM (2017). Transcriptional and post-transcriptional gene regulation by long non-coding RNA. Genomics Proteomics Bioinformat.

[19] Salmena L (2011). A ceRNA hypothesis: the Rosetta Stone of a hidden RNA language?. Cell.

[20] Chan JJ (2018). Noncoding RNA:RNA regulatory networks in cancer. Int J Mol Sci.

[21] Xiao Y (2019). LncRNA MIR4435-2HG is downregulated in osteoarthritis and regulates chondrocyte cell proliferation and apoptosis. J Orthop Surg Res.

[22] Li Z (2019). Integrative analysis of DNA methylation and gene expression profiles identifies MIR4435-2HG as an oncogenic lncRNA for glioma progression. Gene.

[23] Huang CY (2015). miR-128-3p suppresses hepatocellular carcinoma proliferation by regulating PIK3R1 and is correlated with the prognosis of HCC patients. Oncol Rep.

[24] Zhao L (2018). Tumor suppressor miR-128-3p inhibits metastasis and epithelial-mesenchymal transition by targeting ZEB1 in esophageal squamous-cell cancer. Acta Biochim Biophys Sin.

[25] Jin B (2018). Long non-coding RNA SNHG15 promotes CDK14 expression via miR-486 to accelerate non-small cell lung cancer cells progression and metastasis. J Cell Physiol.

[26] Li J (2019). MiR-542-3p, a microRNA targeting CDK14, suppresses cell proliferation, invasiveness, and tumorigenesis of epithelial ovarian cancer. Biomed Pharmacother.

[27] Zhang W (2016). PFTK1 regulates cell proliferation, migration and invasion in epithelial ovarian cancer. Int J Biol Macromol.

